# Assessing Sleep hours in Participants with Alzheimer’s Disease: Findings from a Phase I MAD Study of IGC‐AD1

**DOI:** 10.1002/alz.095667

**Published:** 2025-01-09

**Authors:** Margarita Venegas, Maria Alejandra Tangarife, Laura Delgado‐Murillo, Evelyn Gutiérrez, María Paula Naranjo, Jagadeesh S Rao, Saadia Shahnawaz, Laura Tatiana Sánchez, Varduhi Ghazaryan, Fabio Camargo, William Julio De Jesus, Elliot Hong, Claudia Grimaldi, Ram Mukunda, Maria Juanita Arbelaez

**Affiliations:** ^1^ IGC Pharma, Bogotá, Bogotá D.C. Colombia; ^2^ IGC Pharma, Bogota, Bogota Colombia; ^3^ IGC Pharma, Potomac, MD USA; ^4^ Santa Cruz Behavioral, Bayamon, PR USA; ^5^ UTHealth Houston McGovern Medical School, Houston, TX USA

## Abstract

**Background:**

Sleep disturbances are prevalent in Alzheimer’s disease (AD), impacting cognition and quality of life. Trials have also shown increased subjective sleepiness with melatonin doses compared to placebo. High melatonin doses may increase drowsiness, headache, and dizziness, as indicated by a systematic review. A Phase I trial assessed the safety and tolerability of IGC‐AD1, comprising delta‐9 tetrahydrocannabinol (THC) and melatonin. Preliminary findings suggest IGC‐AD1’s safety, with fewer reports of somnolence and dizziness than placebo and no increase in sleep hours. Concerns about melatonin’s sedative properties are addressed through sleep duration assessment.

**Method:**

Data is presented from a Phase 1 Multiple Ascending Dose MAD trial. Caregivers reported participant sleep hours and nocturnal awakenings during daily safety calls. Average sleep hours for each cohort were computed, calculating nightly averages over the 14‐day drug intake period, and then by cohort and treatment group (1 ml for cohort 1, 2 ml for cohort 2, and 3 ml for cohort 3).

**Result:**

Variations in average sleep duration across cohorts were observed. Cohort 1 showed average sleep hours of 8.97 (active) and 8.77 (placebo), while cohort 2 exhibited averages of 8.11 (active) and 9.43 (placebo). Cohort 3 displayed averages of 7.90 (active) and 9.24 (placebo). These findings suggest that the administration of IGC‐AD1 did not meet expectations regarding increased sleep duration, despite containing melatonin.

**Conclusion:**

Although exogenous melatonin is often associated with sedation, the study indicates that IGC‐AD1, containing melatonin, did not lead to the anticipated increase in sleep duration. Further research is needed to understand the underlying mechanisms and optimize therapeutic interventions for sleep disturbances in AD patients.

